# Discordance between self-reported and performance-based function among knee osteoarthritis surgical patients: Variations by sex and obesity

**DOI:** 10.1371/journal.pone.0236865

**Published:** 2020-07-30

**Authors:** Jessica M. Wilfong, Elizabeth M. Badley, J. Denise Power, Rajiv Gandhi, Y. Raja Rampersaud, Anthony V. Perruccio

**Affiliations:** 1 Health Care & Outcomes Research, Krembil Research Institute, University Health Network, Toronto, Ontario, Canada; 2 Arthritis Program, Krembil Research Institute, University Health Network, Toronto, Ontario, Canada; 3 Dalla Lana School of Public Health, University of Toronto, Toronto, Ontario, Canada; 4 Department of Surgery, Faculty of Medicine, University of Toronto, Toronto, Ontario, Canada; Monash University, AUSTRALIA

## Abstract

**Background:**

There is currently no standardized method for measuring functional status in knee osteoarthritis (OA) patients, despite that it is one of the top priorities when determining eligibility for total knee arthroplasty (TKA). The purpose of the current investigation was to identify factors associated with discordance between individual self-report and performance-based measures of function for obese and non-obese men and women with knee OA.

**Methods:**

In a cohort of 727 knee OA patients scheduled for TKA, physical function prior to surgery was assessed with the self-reported physical function subscale of the Western Ontario and McMaster Universities Osteoarthritis Index (WOMAC-pf), and the performance-based Timed Up and Go (TUG). Data on sociodemographic characteristics, health status, knee pain intensity, symptomatic joint site count, and pain catastrophizing were collected via questionnaire. The primary outcome was the difference in rescaled score between a participant’s self-report and performance-based measures of function. Multivariable linear regression stratified by sex and obesity status was used to identify factors associated with discordance.

**Results:**

The mean age of participants was 65.5 years and 55% were women. With younger age, self-reported scores indicated increasingly worse function compared to performance-based scores, regardless of sex or obesity status. Among non-obese individuals, greater knee pain intensity was associated with a participant’s self-report score indicating increasingly worse function compared to their performance-based score. For obese women, pain catastrophizing, and number of symptomatic joints were also associated with discordance as was reporting fewer comorbidities.

**Conclusions:**

Physical function may be differentially represented by self-reported and performance-based measures depending on a variety of patient factors. Our findings add to the evidence which suggests both measures should be used when assessing functional status prior to TKA.

## Introduction

Osteoarthritis (OA) is the most common type of arthritis and is characterized by pain and physical disability. While any joint is susceptible, the knee is one of the most common joints affected. Initial treatment options for OA include exercise, weight loss, physiotherapy, analgesics, anti-inflammatory drugs, or intra-articular injections [1]. However, when conservative options fail to mitigate pain and improve function, total knee arthroplasty (TKA) surgery is recommended.

Physical function limitation, particularly in mobility, is one of the key symptoms leading individuals to seek and undergo surgery [2]. Physical function can be assessed using self-report questionnaires, which assess an individual’s perception of their own level of mobility, and by performance-based measures which objectively assess mobility. In clinical and research settings self-report measures are often preferred due to ease of administration and minimal cost. Although self-report and performance-based measures were at one time considered to be interchangeable methods of measuring functional ability, studies have shown only a low-to-moderate correlation between the two [3, 4].

The literature suggests that the two types of measurements provide complementary but distinct information about function [5, 6]. Factors including sociodemographic characteristics, cognitive functioning, and personality characteristics, have been suggested to differentially influence self-report and performance-based measures. For example, chronic pain has been closely linked to self-reported disability [5, 6], but generally no association has been found for performance-based measures [7]. Further, a study by Louie and Ward [8] showed that performance-based measures only account for part of the variation in self-report scores. Other factors, including obesity and sex, have been shown to be associated with both self-reported and performance-based measures of function [9–11], though their effect on the relationship between the two types of measures is not yet understood.

Despite an extensive literature on the use of self-report approaches to measure physical functioning, few studies have examined what contributes to discrepancies between these measures and performance-based measures. While sex and obesity are associated with self-report and performance-based function, they additionally are well-established risk factors for knee OA [12]. Therefore, the analyses in this study were stratified by these factors. Our objective was to determine which factors, in obese and non-obese men and women, are associated with discordance between self-reported and performance-based measures of functional ability in a sample of individuals with knee OA.

## Methods

### Study design and participants

The study sample was a subset of patients from an ongoing prospective cohort study, the Longitudinal Evaluation in the Arthritis Program (LEAP-OA) study, at the Toronto Western Hospital in Toronto, Ontario, Canada. Inclusion criteria for the larger study were 18 years of age or older, ability to read and comprehend English, and undergoing surgical intervention for OA. Exclusion criteria included post-traumatic or inflammatory arthritis and revision surgery. The present study was restricted to patients scheduled for total knee arthroplasty (TKA) surgery and included 727 patients, 326 men and 401 women, recruited between November 2013 and April 2019. The study was approved by the University Health Network Research Ethics Board. Written informed consent was obtained from all patients.

### Data collection

Participants completed questionnaires and a performance-based task within the three-week period prior to their scheduled surgery. Data were collected on sociodemographic characteristics including age, sex, and highest level of education. The American Academy of Orthopedic Surgeon’s Comorbidity scale [13] was used to capture comorbid conditions, with individuals indicating yes/no on a list of 20 health conditions. A comorbidity count was derived from the sum of ‘yes’ responses. Respondents indicated on a homunculus diagram all joints (neck, back, right and left shoulder, elbow, wrist, hand, hip, knee, ankle, and foot) that were “painful, stiff or swollen on most days of the past month”. For analyses, a symptomatic joint site count was derived, including the non-surgical knee, but excluding the surgical knee, and other joints were grouped into sites (i.e., one or both hips constituted 1 site) (range of symptomatic joint site count: 0–10). Pain intensity in the surgical knee was captured using the subscale of the Chronic Pain Grade Scale [14]. Items were scored on an 11-point scale, with responses ranging from 0 (No pain) to 10 (Pain as bad as it could be). The subscale score was calculated as the mean intensity ratings for reported current, worst, and average pain. The Pain Catastrophizing Scale is a 13-item, self-administered questionnaire that measures whether an individual ruminates about their pain, magnifies their pain, and whether they feel helpless to manage their pain [15]. Total scores range from 0 (No catastrophizing) to 52 (Severe catastrophizing). Body mass index (BMI) was calculated (kg/m^2^) using each participant’s measured height and weight. Participants were then categorized as obese (BMI≥30) or non-obese (BMI<30).

Performance-based physical function was assessed using the Timed Up and Go (TUG) test. Participants were instructed to sit back in a standard arm chair and were shown a marker 3 meters away on the floor. They were then instructed to stand up from the chair when the researcher said “Go”, walk to the marker at their normal pace, then turn and walk back to the chair at their normal pace and sit again. The time to complete the task was recorded in seconds. Lower time scores represent fewer functional limitations (i.e. better physical function). Construct validity as well as intra- and inter-tester reliability have been demonstrated for the TUG [16].

Self-reported physical function was assessed using the reliable and valid physical function subscale of the Western Ontario and McMaster Universities Osteoarthritis Index (WOMAC-pf) [17]. The subscale consists of 17 questions to which individuals report the level of difficulty experienced performing specific activities (e.g. going from sit to stand; walking, etc.). Response options range from 0 (None) to 4 (Extreme) for each item and the total score was calculated by summing the responses (possible range: 0–68). Lower scores indicate fewer functional limitations (i.e. better physical function).

### Primary outcome

The primary outcome for the study was the difference between a patient’s score on their self-reported physical function measure (WOMAC-pf) and their performance-based measure of function (TUG). Both the WOMAC-pf scores and TUG scores were rescaled to a 0–100 scale to equate the scales and facilitate comparability. The range for the WOMAC-pf scale is 0–68, therefore the formula used to rescale was (WOMAC-pf– 0)/(68–0)*100. The range of the TUG score in our sample was 6.84–32.13 seconds, and the formula used to rescale was (TUG– 6.84)/(32.13–6.84)*100. The discordance score was the TUG score subtracted from the WOMAC-pf score with zero indicating no difference in an individual’s degree of function as reflected by the two measures. Negative discordance scores resulted if an individual had a lower (i.e. better) self-reported score than their performance-based score. A positive discordance score resulted if an individual had a higher (i.e. worse) self-reported score than their performance-based score.

### Stratification groups

Analyses were performed stratified to elucidate the influence of sex and obesity status on the relationships between the covariates and the discordance score. This resulted in four strata, obese men, obese women, non-obese men, and non-obese women.

### Analysis

Sample descriptive statistics were calculated, and strata differences were assessed using ANOVA and chi-square tests, as appropriate. The correlation between the rescaled TUG score and WOMAC-pf scores was assessed using Pearson correlation coefficient. Multivariable linear regression for each strata was used to identify factors associated with discordance scores between performance-based and self-reported rescaled scores. Multicollinearity among independent variables was assessed by inspection of variance inflation factors; estimates <2.5 were interpreted as indicative of no problematic multicollinearity. P-values <0.05 were deemed statistically significant throughout. For covariate effects appearing to differ in magnitude or direction across strata, statistical tests of interaction were undertaken. For each of the covariates with significant associations we created predictor effect plots to illustrate and interpret regression results. The predicted value of the discordance score was plotted at each value of the covariate, setting all other covariates to their mean values. Analyses were conducted using SAS version 9.4.

### Sensitivity analysis

The WOMAC-pf was restricted to the questions which most closely matched the activities measured by the TUG. For example, degree of difficulty “laying in bed” and “putting on/taking off socks” were removed while 8 of 17 items were retained (“rising from sitting”; “standing”; “walking on flat surface”; “getting in/out of car”; “rising from bed”; “getting in/out of bath”; “sitting”; “getting on/off toilet”). Response options range from 0 (None) to 4 (Extreme) for each item and the total score was calculated by summing the responses (possible range: 0–32). Rescaling and analyses were re-run using this restricted WOMAC-pf score.

## Results

Table 1 presents a description of the study sample by strata. There were 143 obese men, 203 obese women, 183 non-obese men, and 198 non-obese women. The overall age range was 39–91 years and those who were obese were younger than those who were not. Overall, women reported higher knee pain intensity, had worse pain catastrophizing scores, and reported more symptomatic joint sites than men, regardless of obesity status. Non-obese men reported significantly less comorbidities than any other group. Additionally, women tended to have greater functional limitations than men, and obese individuals had greater self-reported and performance-based functional limitations than non-obese individuals.

**Table 1:** Characteristics of the sample by strata.

	Obese		Non-obese	
	Men (n = 143)	Women (n = 203)	Men (n = 183)	Women (n = 198)
	n (%) or mean (±SD)	n (%) or mean (±SD)	n (%) or mean (±SD)	n (%) or mean (±SD)
**Age**	63.7 (±8.2)	61.8 (±8.1)	68.0 (±9.0)	68.3 (±8.9)
**Education**				
High School or less	49 (34.8%)	68 (33.7%)	54 (30.2%)	63 (33.0%)
Post-Secondary	92 (65.2%)	134 (66.3%)	125 (69.8%)	128 (67.0%)
**Knee pain intensity**^a^	6.2 (±2.2)	7.1 (±1.8)	5.8 (±2.0)	6.3 (±2.0)
**Pain catastrophizing**^b^	14.5 (±11.7)	20.2 (±12.9)	12.1 (±9.8)	15.8 (±11.5)
**Symptomatic joint site count**^c^	2.0 (±1.8)	2.9 (±2.4)	1.9 (±1.9)	2.6 (±2.3)
**Comorbidity count**^d^	2.2 (±1.5)	2.6 (±2.0)	1.6 (±1.4)	2.0 (±1.7)
**WOMAC-pf score**^e^	50.7 (±18.1)	57.5 (±16.9)	44.1 (±16.9)	50.5 (±17.2)
**TUG score (seconds)**^e^	26.7 (±16.8)	34.1 (±19.8)	23.1 (±13.7)	29.5 (±18.7)
**Discordance score**^f^	24.0 (±20.0)	23.4 (±21.8)	21.0 (±19.0)	21.0 (±21.6)

^a^range: 0–10;

^b^range: 0–52;

^c^range: 0–10;

^d^range: 0–20;

^e^range: 0–100;

^f^range: -63.8–78.2.

The correlation between TUG and WOMAC-pf rescaled scores was moderate, r = 0.33; 95% CI = 0.26, 0.39; p < .001. While the correlation appeared somewhat weaker among non-obese individuals, r = 0.24; 95% CI = 0.10, 0.37 for men and r = 0.28; 95% CI = 0.15, 0.40 for women, compared to obese individuals, r = 0.34; 95% CI = 0.19, 0.48 for men and r = 0.30; 95% CI = 0.17, 0.42 for women, the differences were not statistically significant. Discordance scores ranged from -63.8 to 78.2. The mean discordance score across all strata was 22.3 and 88% of individuals in the sample had a positive discordance score, indicating that, on average, individuals had a higher (i.e. worse) self-reported score than their performance-based score.

**Fig 1:**
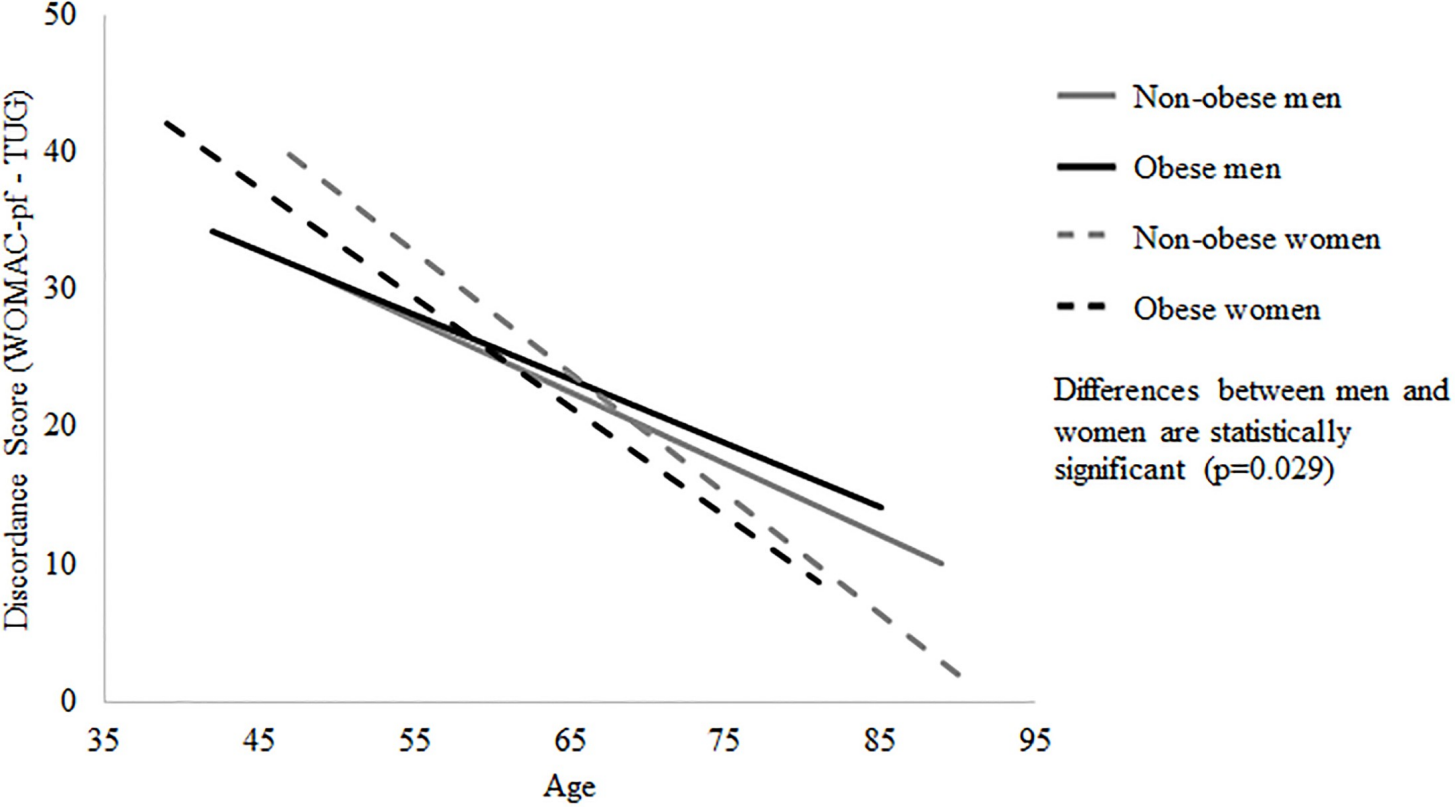
Relationship between age and discordance score. A positive discordance score occurs when the self-report score is higher (i.e. reflects relatively worse function) than the performance-based score. A negative discordance score occurs when the self-report is lower (i.e. reflects relatively better function) than the performance-based score. A discordance score near zero reflects concordance between the two measures.

**Fig 2:**
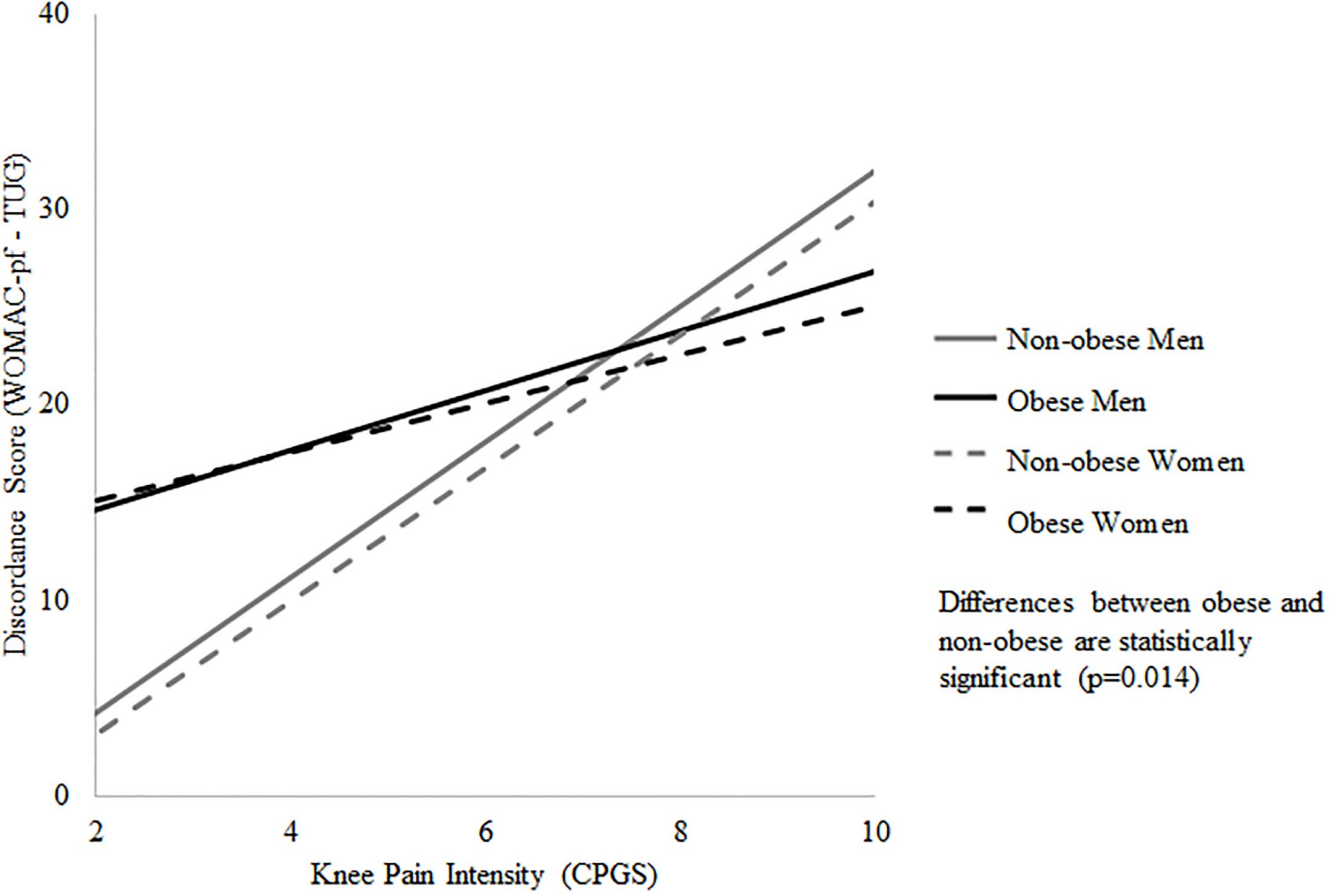
Relationship between knee pain intensity and discordance score. A positive discordance score occurs when the self-report score is higher (i.e. reflects relatively worse function) than the performance-based score. A negative discordance score occurs when the self-report is lower (i.e. reflects relatively better function) than the performance-based score. A discordance score near zero reflects concordance between the two measures.

Table 2 shows the results from the stratified regression analyses. Across strata, older age was negatively associated with a participant’s discordance score. Keeping in mind that a large majority of the sample had positive discordance scores, and as reflected by the model-based predictive plots (Fig 1), the negative association between age and discordance score reflects greater concordance (i.e., smaller positive discordance score) between the self-report and performance-based measures with increasing age. The positive association between knee pain intensity and discordance scores for both non-obese men and non-obese women indicates that increasing knee pain intensity was associated with increasingly worse self-reported functional scores compared to the performance-based scores (Fig 2). For obese women and non-obese men, increasing pain catastrophizing scores were associated with increasingly worse self-reported scores relative to performance-based scores. Finally, among obese women only, increasing joint site count was associated with increasingly worse self-reported scores relative to performance-based scores and increasing comorbidity count was associated with greater concordance between the self-report and performance-based measures. The results of the regression with standardized coefficients are available in S1 Table to allow for comparison between strata.

**Table 2:** Results from the sex- and obesity-stratified linear regression analyses (outcome: Discordance score using WOMAC-pf).

	Obese				Non-obese				
	Men		Women		Men		Women		
	Est	95% CI	Est	95% CI	Est	95% CI	Est	95% CI	p-value for interaction
**Age**	**-0.46**	**-0.84, -0.09**	**-0.79**	**-1.14, -0.44**	**-0.52**	**-0.80, -0.24**	**-0.88**	**-1.18, -0.58**	Age*Sex **0.029**
**Education** (post-secondary vs less)	3.25	-2.96, 9.47	2.85	-3.19, 8.90	-5.23	-10.55, 0.08	-2.53	-8.58, 3.51	Education*Sex* Obesity **0.039**
**Knee pain intensity**	1.31	-0.29, 2.91	1.04	-0.61, 2.68	**3.23**	**1.84, 4.62**	**3.18**	**1.64, 4.72**	Pain*Obesity **0.014**
**Pain catastrophizing**	0.26	-0.02, 0.54	**0.30**	**0.06, 0.54**	**0.32**	**0.05, 0.59**	-0.08	-0.35, 0.19	PCS*Sex*Obesity **0.038**
**Symptomatic joint site count**	-0.18	-1.84, 1.47	**1.34**	**0.09, 2.58**	0.39	-0.87, 1.65	0.99	-0.30, 2.28	Site count*Sex 0.216
**Comorbidity count**	1.40	-0.53, 3.33	**-1.49**	**-2.95, -0.04**	-0.93	-2.77, 0.91	-0.41	-2.17, 1.36	Comorbidity*Sex*Obesity **0.024**

Statistically significant (p<0.05) *P*-values are indicated in bold.

Results from the sensitivity analysis using the restricted set of WOMAC-pf items were highly consistent with those from the primary analysis (Tables 3 and 4). S2 Table provides the results of the regression with standardized coefficients to allow for comparison between strata.

**Table 3:** Restricted WOMAC-pf score and restricted discordance score by strata.

	Obese		Non-obese	
	Men (n = 143)	Women (n = 203)	Men (n = 183)	Women (n = 198)
	n (%) or mean (±SD)	n (%) or mean (±SD)	n (%) or mean (±SD)	n (%) or mean (±SD)
**Restricted WOMAC-pf score**^a^	47.7 (±19.2)	55.2 (±17.4)	40.7 (±17.4)	48.5 (±18.0)
**Restricted discordance score**^b^	21.0 (±21.0)	21.2 (±22.2)	17.6 (±19.8)	19.0 (±22.2)

^a^range: 0–100;

^b^range: -64.9–80.2.

**Table 4:** Results from the sex- and obesity-stratified linear regression sensitivity analyses (outcome: Restricted discordance score).

	Obese				Non-obese			
	Men		Women		Men		Women	
	Est	95% CI	Est	95% CI	Est	95% CI	Est	95% CI
**Age**	**-0.67**	**-1.06, -0.29**	**-0.76**	**-1.12, -0.40**	**-0.53**	**-0.83, -0.23**	**-0.88**	**-1.19, -0.56**
**Education** (post-secondary vs. less)	3.94	-2.48, 10.36	2.95	-3.20, 9.10	-4.62	-10.24, 1.00	-2.02	-8.29, 4.25
**Knee pain intensity**	1.00	-0.66, 2.65	0.79	-0.88, 2.47	**3.00**	**1.53, 4.47**	**3.28**	**1.68, 4.88**
**Pain catastrophizing**	0.24	-0.05, 0.53	**0.30**	**0.05, 0.55**	**0.32**	**0.03, 0.61**	-0.12	-0.4, 0.16
**Symptomatic joint site count**	-0.32	-2.03, 1.39	**1.58**	**0.31, 2.85**	0.80	-0.53, 2.14	1.10	-0.24, 2.44
**Comorbidity count**	1.94	-0.05, 3.94	**-1.81**	**-3.29, -0.32**	-1.09	-3.04, 0.85	-0.57	-2.40, 1.26

Statistically significant (p<0.05) *P*-values are indicated in bold.

## Discussion

In this study, we sought to identify factors associated with discordance between a self-reported and a performance-based measure of function, by sex and obesity status, in a sample of patients scheduled for TKA for knee OA. Performance-based function scores were only moderately correlated with self-reported scores [3, 4]. Using a novel approach by calculating a discordance score, we were able to identify factors influencing individual variations between self-reported and performance-based scores, which to our knowledge has not been previously reported.

Younger age was associated with a self-report score indicating increasingly worse self-reported function scores compared to the performance-based scores while older age was associated with greater concordance between the two measures. In our sample, we found that age was significantly negatively associated with WOMAC-pf score. A few studies of knee OA which include middle-aged participants have found a similar association between age and self-reported physical function [18, 19]. Specifically, one study by Gignac et al., comparing the perception of osteoarthritis-related experiences between middle-aged and older-age adults, found that younger individuals were more upset by their OA than older respondents mainly because the disease was not perceived as normative in their age group [18]. Additionally, the authors noted that those under the age of 65 tend to be engaged in more activities (e.g., working, activities with young children) which may be disrupted by OA symptoms, causing additional distress. In younger age groups, these experiences and perceptions may be reflected by a pattern of worse self-reported dysfunction compared to performance-based function.

Among both non-obese men and women there was a positive association between knee pain intensity and the discordance score such that higher knee pain was associated with increasingly worse self-reported functional scores compared to the performance-based scores. This finding is supported by studies which have suggested that chronic pain is strongly associated with lower self-reported function but generally has no association with performance-based function [5, 6]. However, it was surprising that a similar association was not found among obese individuals. A study examining the correlation between self-report and performance-based measures reported a lower correlation in patients with greater pain [20]. As obese women reported significantly more pain than any other group in this study, we would have expected to find an association between pain and discordance for this group. Disablement process theory suggests that the way individuals respond to their disablement experience during daily activities depends on their expectations of what they are capable of doing [21, 22]. Therefore, it is surmised that while knee pain may cause some decreased walking ability for both obese and non-obese individuals, non-obese individuals self-rate their limitations higher than obese individuals who may have already adapted to having more difficulty walking.

There are conflicting findings in the literature regarding the relationship between pain catastrophizing and performance-based function. While some have found that those who pain catastrophize tend to have worse performance-based physical function [23, 24], others have found no association [25]. In the study which found no association, the authors theorized that their conflicting findings could stem from differing patient characteristics and/or pain phenotypes in their study population compared to others. Our finding that only among obese women and non-obese men increasing pain catastrophizing was associated with greater discordance supports this theory. It is likely that pain catastrophizing has differing effects on physical performance depending on patient characteristics, including sex and obesity status, though more work is needed to better elucidate what factors play the most important roles in this complex relationship.

When completing the WOMAC-pf questionnaire patients were instructed to think of their knee problems. However, the daily activities referenced in the questionnaire involve joints other than the knee alone. Others have reported poorer functional outcomes with multi-joint symptoms [26]. For this reason, the number of symptomatic joint sites (other than the surgical knee) was considered in our investigation. As expected, a higher number of symptomatic joints was associated with increasingly worse self-reported function relative to the performance-based measure. However, this discordance was significant in obese women only. Prior work in a sample of TKA patients found that higher symptomatic joint burden was associated with greater systemic inflammatory load in women but not men [27]. Additionally, findings of positive associations between obesity and OA in non-weight-bearing joints also suggest systemic components of OA [28–30]. Increased systemic inflammatory load may to some extent contribute to overall poorer functional performance [31], and may explain the sex and obesity differences reported here.

Finally, we also found an association between comorbidity and discordance among obese women only, such that reporting fewer comorbid conditions was associated with having increasingly worse self-reported function compared to the performance-based function, and more comorbid conditions with greater concordance. As noted above, the WOMAC-pf prompts respondents to answer the questions in relation to their knee problems. It is possible that those who have knee OA and few to no other conditions attribute most, if not all, of their physical dysfunction to their knee OA, resulting in worse self-reported scores relative to physical performance score, while for those with several other conditions, in addition to their OA, the attribution may not be as focused on the knee. The strong similarities between the findings from our primary and sensitivity analyses allow us to conclude that differences in the nature of self-report and performance-based measures likely explain the results, not simply differences between the two specific measures chosen for the current study.

A strength of this study is that it investigated the influence of a number of factors on the discordance between performance-based and self-report measures, which has not previously been reported. An additional strength is our consideration of sex- and obesity-specific influences on the discordance between the two types of measures, particularly as others have simply adjusted for sex and obesity in their analyses, an approach that may miss important differences in underlying contributors to self-reported and performance-based scores. Limitations of the study include the restriction to one performance-based task and one self-report measure, which may limit the generalizability of our findings. Additionally, our sample size did not permit stratifying BMI beyond obese and non-obese which may be masking differences between normal and overweight individuals. Further, the sample was limited to those with advanced knee OA scheduled for surgery. Whether findings are generally applicable across the spectrum of disease severity remains to be studied.

Functional status is one of the top priorities when determining eligibility for TKA along with pain, structural damage [2], and patient quality of life [32]. Currently there is no standardized tool for surgeons to assess functional status prior to TKA. Overall, our findings add to the literature that suggests that performance-based and self-report measures of function provide different, but complementary information about physical function. Ideally these should be used in conjunction with each other to gain a more holistic picture of an individual’s physical function. As many surgeons rely on self-report measures for convenience, and particularly when resources and time are limited, our findings suggest that greater awareness is needed of the differences in how functional difficulties are represented by self-reported and performance-based measures of function prior to TKA surgery for OA. 

## Supporting information

S1 Table Standardized results from the sex- and obesity-stratified linear regression analyses (outcome: Discordance score using WOMAC-pf). (DOCX)

S2 Table Standardized results from the sex- and obesity-stratified linear regression sensitivity analyses (outcome: Restricted discordance score). (DOCX)
