# Supplementary material for: Discordance between self-reported and performance-based function among knee osteoarthritis surgical patients: Variations by sex and obesity
Source: PLoS One. 2020 Jul 30;15(7):e0236865. doi: 10.1371/journal.pone.0236865 (PMC7392249; doi:10.1371/journal.pone.0236865)
Supplement: S1 Table — (DOCX) [file pone.0236865.s001.docx]

**Supplementary Table 1. Standardized results from the sex- and obesity-stratified linear regression analyses (outcome: discordance score using WOMAC-pf).**

|  | **Obese** | | | | **Non-obese** | | | |
| --- | --- | --- | --- | --- | --- | --- | --- | --- |
|  | **Men** | | **Women** | | **Men** | | **Women** | |
|  | **Std Est** | **95% CI** | **Std Est** | **95% CI** | **Std Est** | **95% CI** | **Std Est** | **95% CI** |
| **Age** | **-0.20** | **-0.37, -0.04** | **-0.34** | **-0.50, -0.19** | **-0.22** | **-0.35, -0.10** | **-0.38** | **-0.52, -0.25** |
| **Education**  (post-secondary vs less) | 0.07 | -0.07, 0.21 | 0.06 | -0.07, 0.20 | -0.12 | -0.24, 0.00 | -0.06 | -0.19, 0.08 |
| **Knee pain intensity** | 0.13 | -0.03, 0.29 | 0.10 | -0.06, 0.26 | **0.32** | **0.18, 0.45** | **0.31** | **0.16, 0.46** |
| **Pain catastrophizing** | 0.15 | -0.01, 0.31 | **0.17** | **0.03, 0.31** | **0.18** | **0.03, 0.34** | -0.05 | -0.20, 0.11 |
| **Symptomatic joint site count** | -0.02 | -0.19, 0.16 | **0.14** | **0.01, 0.27** | 0.04 | -0.09, 0.17 | 0.10 | -0.03, 0.24 |
| **Comorbidity count** | 0.12 | -0.04, 0.28 | **-0.12** | **-0.25, 0.00** | -0.08 | -0.23, 0.08 | -0.03 | -0.18, 0.11 |

Statistically significant (p<0.05) *P*-values are indicated in bold.
