# Supplementary material for: Discordance between self-reported and performance-based function among knee osteoarthritis surgical patients: Variations by sex and obesity
Source: PLoS One. 2020 Jul 30;15(7):e0236865. doi: 10.1371/journal.pone.0236865 (PMC7392249; doi:10.1371/journal.pone.0236865)
Supplement: S2 Table — (DOCX) [file pone.0236865.s002.docx]

**Supplementary Table 2. Standardized results from the sex- and obesity-stratified linear regression sensitivity analyses (outcome: restricted discordance score).**

|  | **Obese** | | | | **Non-obese** | | | |
| --- | --- | --- | --- | --- | --- | --- | --- | --- |
|  | **Men** | | **Women** | | **Men** | | **Women** | |
|  | **Std Est** | **95% CI** | **Std Est** | **95% CI** | **Std Est** | **95% CI** | **Std Est** | **95% CI** |
| **Age** | **-0.28** | **-0.45, -0.12** | **-0.32** | **-0.47, -0.17** | **-0.22** | **-0.35, -0.10** | **-0.37** | **-0.50, -0.24** |
| **Education** (post-secondary vs. less) | 0.09 | -0.05, 0.23 | 0.06 | -0.07, 0.20 | -0.10 | -0.22, 0.02 | -0.04 | -0.18, 0.09 |
| **Knee pain intensity** | 0.09 | -0.06, 0.25 | 0.08 | -0.08, 0.23 | **0.29** | **0.15, 0.42** | **0.31** | **0.16, 0.46** |
| **Pain catastrophizing** | 0.13 | -0.03, 0.29 | **0.17** | **0.03, 0.31** | **0.18** | **0.02, 0.34** | -0.07 | -0.22, 0.09 |
| **Symptomatic joint site count** | -0.03 | -0.21, 0.14 | **0.16** | **0.03, 0.29** | 0.08 | -0.05, 0.22 | 0.11 | -0.02, 0.25 |
| **Comorbidity count** | 0.16 | 0.00, 0.32 | **-0.15** | **-0.27, -0.03** | -0.09 | -0.25, 0.07 | -0.05 | -0.19, 0.1 |

Statistically significant (p<0.05) *P*-values are indicated in bold.
